# The roles of signal transducer and activator of transcription factor 3 in tumor angiogenesis

**DOI:** 10.18632/oncotarget.19932

**Published:** 2017-08-04

**Authors:** Peng Gao, Na Niu, Tianshu Wei, Hideto Tozawa, Xiaocui Chen, Caiqing Zhang, Jiandong Zhang, Youichiro Wada, Carolyn M. Kapron, Ju Liu

**Affiliations:** ^1^ Medical Research Center, Shandong Provincial Qianfoshan Hospital, Shandong University, Jinan, Shandong, China; ^2^ Department of Pediatrics, Shandong Provincial Hospital Affiliated to Shandong University, Jinan, Shandong, China; ^3^ The Research Center for Advanced Science and Technology, Isotope Science Center, The University of Tokyo, Meguro-ku, Tokyo, Japan; ^4^ Department of Respiratory Medicine, Shandong Provincial Qianfoshan Hospital, Shandong University, Jinan, Shandong, China; ^5^ Department of Radiation Oncology, Shandong Provincial Qianfoshan Hospital, Shandong University, Jinan, Shandong, China; ^6^ Department of Biology, Trent University, Peterborough, Ontario, Canada

**Keywords:** STAT3, tumor angiogenesis, transcriptional regulation, endothelial cells

## Abstract

Angiogenesis is the development of new blood vessels, which is required for tumor growth and metastasis. Signal transducer and activator of transcription factor 3 (STAT3) is a transcription factor that regulates a variety of cellular events including proliferation, differentiation and apoptosis. Previous studies revealed that activation of STAT3 promotes tumor angiogenesis. In this review, we described the activities of STAT3 signaling in different cell types involved in angiogenesis. Particularly, we elucidated the molecular mechanisms of STAT3-mediated gene regulation in angiogenic endothelial cells in response to external stimulations such as hypoxia and inflammation. The potential for STAT3 as a therapeutic target was also discussed. Overall, this review provides mechanistic insights for the roles of STAT3 signaling in tumor angiogenesis.

## INTRODUCTION

Angiogenesis is defined as the development of new capillaries from pre-existing vessels [1, 2]. Physiological angiogenesis is crucial for embryo development, wound healing, and ovarian cycle. Pathological angiogenesis is associated with various diseases, including diabetes, arthritis, macula degeneration and cancer [3, 4]. As solid tumor requires new vessels to supply the blood with nutrients and oxygen, tumor angiogenesis is essential for tumor growth and became a key target for chemotherapy [5, 6].

Generally, when the diameter of a tumor is larger than 2 millimeters, the tissue penetration can no longer sustain tumor growth, resulting in hypoxia in the microenvironment [7, 8]. In hypoxic condition, tumor cells produce and secrete pro-angiogenic cytokines, which activate endothelial cells (ECs), the inner layer of the existing blood vessels [9, 10]. The pro-angiogenic cytokines include basic fibroblast growth factor (bFGF), vascular endothelial growth factor (VEGF), and platelet-derived growth factor (PDGF) [11–13], all of which have specific receptors on endothelial cells [14]. Upon activation by growth factor-receptor interaction, endothelial cells sprout by migration and proliferation [15]. Tumor cells and ECs both secrete matrix metalloproteinases (MMPs), which degrade basement membranes and the extracellular matrix (ECM) connecting with ECs [16]. The primary sprouts form tubes and then capillary loops, followed by recruitment of pericytes, synthesis of a new basement membrane and vessel maturation [17]. Tumor vasculature usually lacks of pericytes and intact basement membrane structure, which is convenient for additional tumor angiogenesis and metastasis [18].

VEGF is the most potent pro-angiogenic factor [19]. The expression of VEGF is controlled by cascades of cell signaling events and transcriptional factors. Signal transducer and activator of transcription factor 3 (STAT3) is one of the angiogenesis-related transcription factors [20]. In many types of tumors, activation of STAT3 increases the expression of VEGF and MMPs, promoting tumor angiogenesis. In endothelial cells, STAT3 transduces VEGF signals, and promotes EC proliferation, migration, and survival. Targeting STAT3 for tumor angiogenesis becomes a promising strategy. In this review, we will focus on the roles of STAT3 in mediating the expression of angiogenesis-related genes and discuss the molecular mechanisms behind STAT3-regulated tumor angiogenesis.

### STAT family members

**Figure 1:**
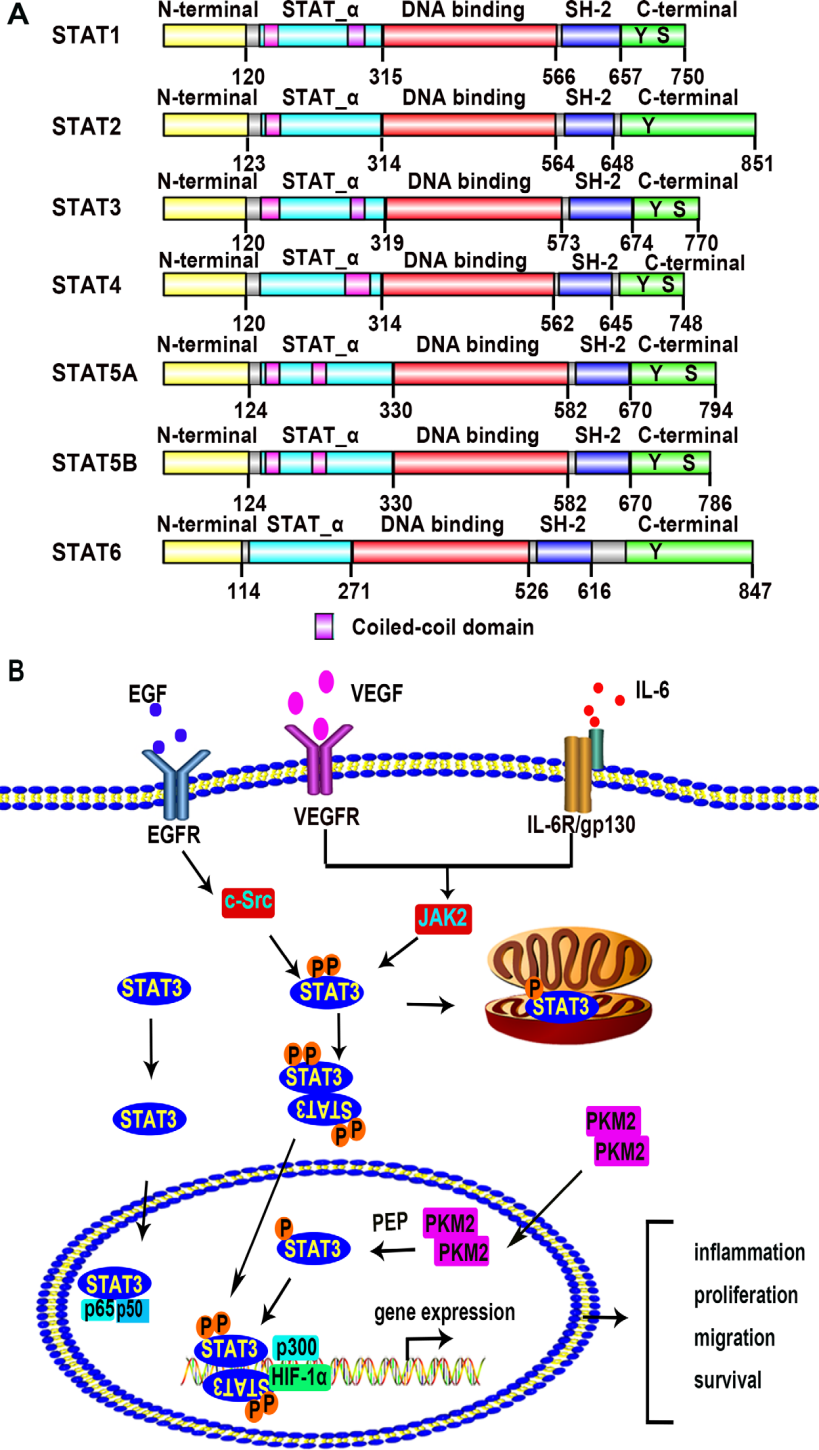
The STAT family members and the STAT3 signaling pathway (**A**) A conceptual diagram showing the structure of STAT family members. There are seven members in the STAT family including STAT1, STAT2, STAT3, STAT4, STAT5A, STAT5B, and STAT6. STAT family members are sharing five highly homologous domains including a N-terminal domain, an STAT_α domain, a DNA-binding domain, an SH2 domain, and a C-terminal domain with transactivation activity. There are some amino acid residues which are crucial for STAT dimerization and transactivation, such as tyrosine 705 (dimerization and DNA binding) and serine 727 (transactivation) residue. These data come from the database of Pfam which belongs to the European Molecular Biology Laboratory-The European Bioinformatics Institute (EMBL-EBI). (http://pfam.xfam.org/protein/P42224/P52630/P40763/Q14765/P42229/P51692/P42226). (**B**) Schematic depiction of the STAT3 signaling pathway. Extracellular signals including growth factors and cytokines activate STAT3. Binding of these signal factors to their cell surface receptors results in phosphorylation of STAT3 at tyrosine 705 and serine 727. STAT3 monomers then dimerize, binding with each other at the SH2 domain. After translocation into the nucleus, the dimerized STAT3 molecule binds to the promoter region of target genes with or without co-factors, modulating transcription of the genes related to inflammation, cellular proliferation, migration, survival. Unphosphorylated STAT3 monomers also translocate into the nucleus and form a complex with p65 and p50 to regulate NF-κB signal pathway. PKM2 dimers act as a protein kinase to phosphorylate STAT3 at tyrosine 705 in the nucleus.

The STAT family includes 7 members: STAT1, STAT2, STAT3, STAT4, STAT5A, STAT5B, and STAT6; all of which act as transcription factors [20–22]. STAT family members share five highly homologous domains including a N-terminal domain, an STAT_α domain, a DNA-binding domain, an Src homology 2 (SH2) domain, and a C-terminal domain with transactivation activity (Figure 1A). The human *Stat3* gene is located on chromosome 17q21.2 [23]. STAT3 protein is composed of 770 amino acids with a molecular weight of 92 kilo-dalton (KDa). There are two phosphorylation sites tyrosine 705 and serine 727 on the C-terminal domain of STAT3. The tyrosine is necessary for STAT activation, and the serine phosphorylation occurs after tyrosine phosphorylation. The serine site of STAT3 might be phosphorylated alone, but this phosphorylation does not configure STAT3 transactivation [23].

### STAT3 signal transduction and STAT3-regulated transcription

The activation of STAT3 is initiated by the binding of growth factors and cytokines with their receptors. As shown in Figure 1B, VEGF and epidermal growth factor (EGF) bind to their receptors and promote the dimerization of receptors [24, 25]. VEGFR and EGFR are both receptor tyrosine kinases (RTKs). The dimerized receptors auto-phosphorylate each other and then phosphorylate the non-receptor protein tyrosine kinases janus kinase 2 (JAK2) and c-Src kinase. The JAK2 and c-Src kinase in turn phosphorylate STAT3 at tyrosine 705, leading to STAT3 dimer or formation and translocation into the nucleus [26]. The serine 727 may also be phosphorylated by mitogen-activated protein kinase (MAPK) and protein kinase C (PKC), but it is not as essential as the tyrosine 705 for STAT3 activation [27]. The interleukin-6 (IL-6) signal also transduce though JAK2 as shown in Figure 1B. The receptor of IL-6 is a complex which contains IL-6 receptor subunit (IL6R) and IL-6 signal transducer glycoprotein 130 (gp130). The complex activates STAT3 in a JAK2 dependent way [28]. The phosphorylated STAT3 translocates into nucleus and regulates the target genes by recognizing specific DNA sequences known as the interferon -gamma activated sequence (GAS), TTCnnnGAA [26, 29]. While most STATs proteins require phosphorylation and dimerization before transport into nucleus, STAT3 may translocate into the nucleus as a monomer. Once entering into the nucleus, monomeric aphosphotyrosine STAT3 (STAT3_aPTyr_) binds to p65 (RELA) and p50, and the complex competitively inhibits the binding of NF-κB p65 homodimer to its targeted DNA elements, thus preventing NF-κB mediated gene expression during the acute-phase response [30]. Other STAT family members do not inhibit NF-κB targeted gene expression. In addition, STAT3 exists in mitochondria of mature central nervous system (CNS) neurons and provides an ancillary role for axon re-growth [31]. In mouse embryonic fibroblasts (MEF), STAT3 reduces reactive oxygen species (ROS) production by binding to cyclophilin D (CypD) in the mitochondria [32].

### Modulation of STAT3 activation

Recent findings suggest that pyruvate kinase M2 (PKM2), a glycolysis enzyme, directly activates STAT3 [33, 34]. PKM2 catalyzes phosphoenolpyruvate (PEP) to pyruvate in a tetramer form in both aerobic and anaerobic glycolysis [35]. In tumor cells, the dimeric PKM2 is the main form of existence [36–38]. The PKM2 dimers act as a protein kinase to phosphorylate STAT3 at tyrosine 705 where PEP is the donor to provide phosphate [34]. The nuclear localization signal (NLS) of PKM2 facilitates its translocation into the nucleus, thus is essential for PKM2 mediated STAT3 activation [37]. The PKM2/STAT3 pathway promotes cell proliferation and migration [39, 40]. In colorectal cancer (CRC) cells, inhibition of STAT3 will significantly restrict PKM2 induced cytokines production and cell proliferation [40]. In liver cancer cells, over-expression of PKM2 increases proliferation and transfection with siRNA of PKM2 decreases proliferation, while phosphorylation of STAT3 at Y705 but not S727 is enhanced by PKM2 [41]. PKM2 is also reported to be interacting with STAT3 to up-regulate *Mek5* expression [35, 42]. Over-expression of PKM2 up-regulates MMP2, MMP9, and N-cadherin, while inhibition of STAT3 abolishes these effects of PKM2 [39].

In addition to the canonical and non-canonical phosphorylation of STAT3, acetylation of STAT3 is a post-translational modification of STAT3 in response to cytokine stimulation [43]. Oncostatin M (OSM), IL-6, and interferon-α (IFN-α) are all reported to increase STAT3 acetylation by promoting the interaction of histone acetyltransferase (HAT) with STAT3 [29, 44]. Nie et al. confirmed that acetylation of the C-terminal lysine 679, 685, 707, and 709 lysine sites are associated with STAT3 phosphorylation, which is not affected by acetylation of the N-terminal 49 and 87 lysine sites [45]. Site-mutagenesis of the four C-terminal acetylation sites of STAT3 together inhibits STAT3 phosphorylation at site tyrosine 705, and the nuclear localization and transcriptional activity of STAT3 are also suppressed [45]. Thus, phosphorylation of STAT3 at site tyrosine 705 requires C-terminal lysine acetylation.

### Phenotypes of STAT3 deletion mouse models

STAT3 is required during embryonic development. Two STAT3 isoforms were generated by alternatively splicing, the full-length STAT3α and the C-terminal activation domain truncated STAT3β with a shift from TTCSNTI to FIDAVWK, corresponding to amino acids 716–722 [46, 47]. Germline *Stat3αβ* deletion leads to embryonic degeneration and lethality after egg cylinder stage between E6.5 and E7.5 [48]. Deletion of *Stat3α* does not cause embryonic lethality, but the newborn *Stat3α^−/−^* mice die afterbirth due to dyspnea. Mice lacking *Stat3β* survive to adulthood; however, they are hypersensitive to lipopolysaccharide (LPS) -induced inflammation. The expression of STAT3β rescues embryonic deaths caused by *Stat3αβ*-null mutation [49, 50]. STAT3β is not a dominant negative factor as it mediates the transcription of acute-phase genes in the liver [49].

To delete *Stat3* gene in specific tissues or cells *in vivo*, the conditional knockout models of *Stat3* have been generated and crossed with tissue specific Cre mice. Mice with conditional STAT3 deletions in ECs are healthy and fertile, but their mortality after LPS treatment is increased [51]. STAT3-deletion in cardiac ECs decreases STAT3 activity in cardiomyocytes, leading to a decreased recovery of myocardial function after ischemia/reperfusion (I/R). These affected cardiomyocytes showed higher levels of caspase-8 activity, and underwent apoptosis after I/R [52, 53]. At the same time, myocardial expressions of (Interleukin-6) IL-6 and suppressor of cytokine signal 3 (SOCS3) are increased after I/R, suggesting that STAT3-null cardiac ECs may not receive the pro-angiogenic signal released by cardiomyocytes. On the other hand, the deletion of STAT3 in cardiomyocytes inhibits EC proliferation [54], perhaps as a result of enhanced expression of anti-angiogenesis factors, such as tissue inhibitor of metalloproteinase 1, connective tissue growth factor, and thrombospondin-1 [55–58]. Therefore, both cardiomyocytes and ECs require STAT3 activation to promote cardiac angiogenesis, and STAT3 may mediate the crosstalk between cardiomyocytes and ECs. STAT3 also participates in hypoxia-induced angiogenesis. STAT3 ablation in mesenchymal stem cells (MSCs) neutralizes hypoxia-induced release of VEGF and inhibits angiogenesis after ischemia, which decreases the protective role of MSCs against myocardial ischemia [59]. STAT3 deficiency in the pancreas causes incomplete development of the pancreatic microvasculature, which may restrict insulin transportation, leading to glucose intolerance and a defect in insulin secretion *in vivo* [60]. Together, STAT3 deletion impairs tissue vasculature, and STAT3 promotes angiogenesis by regulating the phenotypes of both ECs and non-ECs.

### Mechanistic events of STAT3 signaling in tumor cells and ECs in angiogenesis

**Figure 2:**
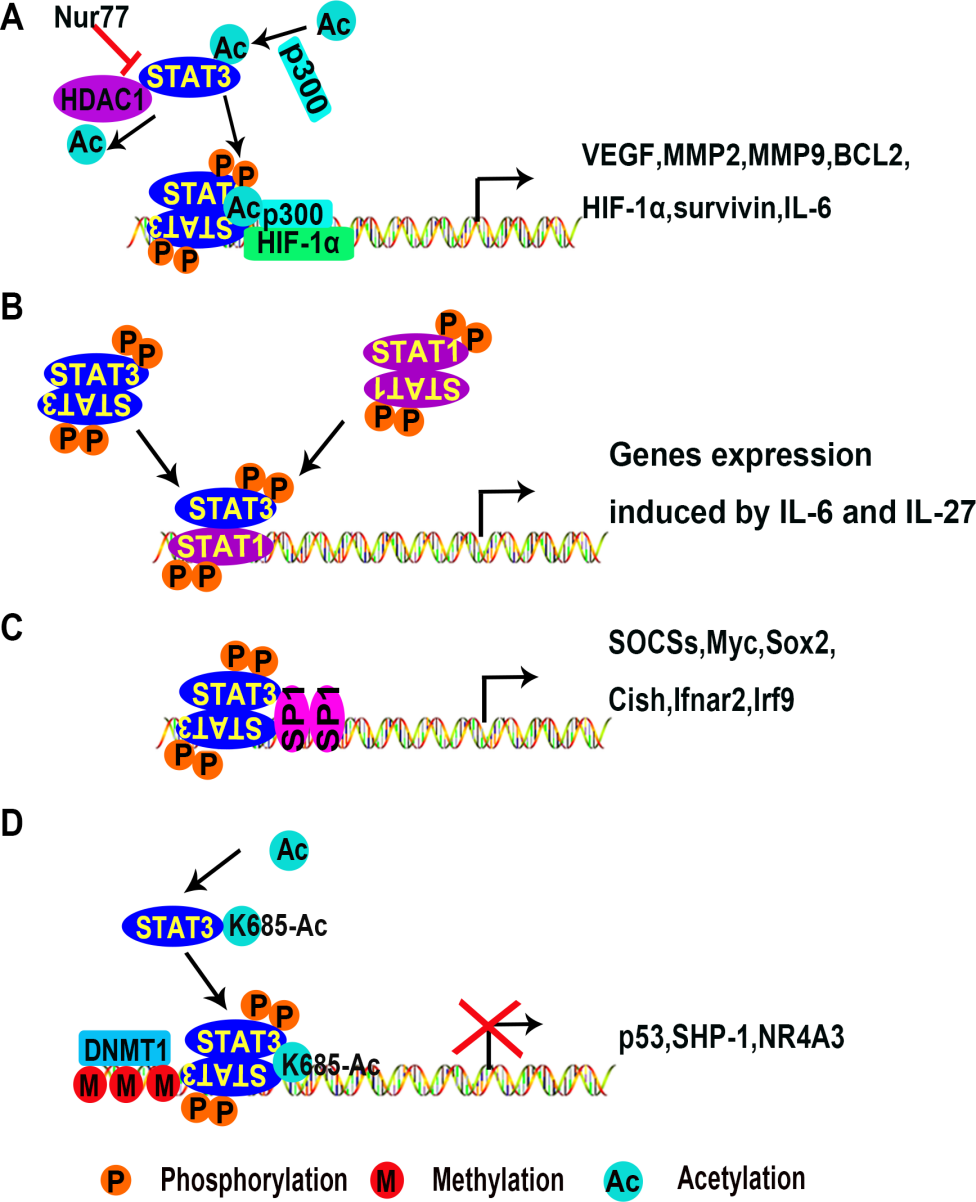
Mechanisms of transcriptional activation or inhibition of target genes by STAT3 (**A**) STAT3 dimers activate gene expression with co-factors p300 and HIF-1α. (**B**) STAT3 is activated by IL-6 and IL-27 in T cells. STAT3 homodimers or heterodimers are translocated into the nucleus and bind to special DNA sequences, initiating gene transcription. (**C**) STAT3 interacts with co-factors such as STAT1 or SP1 to promote gene expression in stem cells. (**D**) STAT3 dimers inhibit gene expression by recruiting the DNA methyltransferase DNMT1. (Ac, acetylation; M, methylation; P, phosphorylation; DNMT1, DNA (cytosine-5)-methyltransferase 1; HDAC1, histone deacetylase 1; HIF-1α, hypoxia-inducible factors 1alpha; MMP2, metalloprotease 2; Nur77, nerve growth factor-induced gene B; SHP-1, Src homology region 2 domain-containing phosphatase 1; SP1, specificity protein 1).

STAT3 engages hypoxia-responsive gene transcription in tumor cells [61]. As shown in Figure 2A, hypoxia induces STAT3 activation and formation of transcriptional complexes that promotes the expression of VEGF in tumors. In pancreatic and prostate carcinomas, such complexes contain STAT3, HIF-1α, CREB-binding protein (CBP/p300), and redox effector factor-1/apurinic/apyrimidinic endonuclease (Ref-1/APE) [62]. Similarly, in breast cancer cells and Hep3B hepatoma cells, complexes constituted by STAT3, CBP/p300, RNA polymerase II (Pol II), and HIF-1α are reported to regulate HIF-1α target genes including VEGF [63]. These STAT3-mediated gene expressions in cancer cells promote angiogenesis. VEGF expression correlates with STAT3 activity in diverse human cancer cell lines. In human cancer cell lines, an activated STAT3 mutant (STAT3C) increases VEGF expression by binding to *VEGF* gene promoter [64]. Inhibiting JAK2 and STAT3 expression both suppressed STAT3 activation and reduced the expression of VEGF and bFGF in non-small-cell lung cancer (NSCLC) [65]. In addition, IL-6 promotes angiogenesis and cervical tumor growth by activating the STAT3-VEGF pathway [66]. Glycophosphatidyl inositol (GPI)-anchored folate receptor alpha (FRalpha) is up-regulated in many types of cancer cells. Folic acid and folinic acid activate STAT3 through the FRalpha/JAK/STAT3 pathway, promoting HeLa cell proliferation through the expression of STAT3 target genes including Cyclin A2 and VEGF [67]. MMP2, MMP9 are also secreted by cancer cells in a STAT3-dependant manner [68, 69]. In addition to direct activation of STAT3, other mechanisms promoting tumor angiogenesis are facilitated by STAT3. For instance, reversion-inducing cysteine-rich protein with kazal motifs (RECK) inhibits STAT3 activity by binding with β1-integrin, IL-6RA, glycoprotein 130 (gp130), and urokinase receptor (uPAR) in the membrane, resulting in remarkable STAT3 inhibition in breast cancer cells. The inhibition of RECK, however, promotes angiogenesis and metastasis of breast cancer [70]. Recent research has found that cigarette smoke extract activates STAT3 in bronchial epithelial cells and promotes exosomal bubbling. The miR-21-containing exosomes are endocytosed by ECs to modulate gene expression and facilitate vascular formation [71]. The miR-9 is secreted by tumor cells, and activates STAT3 in ECs by inhibiting SOCS5 levels, leading to migration of ECs and tumor angiogenesis [72]. These studies demonstrate that the activation of STAT3 in tumor cells establishes a microenvironment that is conducive to angiogenesis by mediating the secretion of pro-angiogenic factors.

The intercellular communication between tumors and ECs is facilitated by STAT3 [73]. ECs are the primary targets of VEGF signal produced by other cells, especially by cancer cells [74]. STAT3 activation mediates VEGF-induced EC migration and tube formation [75, 76]. The IL-6/IL-6R/STAT3 axis is another important pathway for angiogenesis. It is well established that IL-6 activates STAT3 through IL-6Ralpha and gp130 [77]. The L1 transmembrane glycoprotein (also known as L1CAM or CD171) induces STAT3 activity via the IL-6/IL-6RA axis, and promotes cell proliferation, migration, tubulogenesis and vascular permeability in lung ECs [78]. In ECs, STAT3 requires co-factors to promote gene expression. It is reported that HIF-1α, STAT3, and specificity protein 1 (Sp1) can increase VEGF expression in mouse cerebral endothelial cells. To date it is not clear whether these three factors directly interact each other. However, the results of ChIP-PCR showed that the binding site of HIF-1α (−931 to −912) and STAT3 (−805 to −786) are very close, and the Sp1 binding site (−89 to −65) is at the 3′ side. Considering the spatial structure of proteins and DNA, it is possible that these proteins may form a complex and regulate gene expression together [79].

STAT3 also mediates pro-angiogenic signaling events in other cell types. For example, activation of STAT3 can be observed in VSMCs, macrophages, dendritic cells, bone marrow stem cells, and fibroblasts. STAT3 mediates the expression of a large number of genes upon different stimulations in multiple cell types (Table 1). Expression of these downstream genes may be anti-apoptotic or control the cell cycle, cell adhesion or cell migration, thus contributing to angiogenesis [70, 80–99].

**Table 1:** STAT3 regulated genes

Cell types	Target genes	Functions	References
Cancer cells	NR4A3, Forkhead BoxM1(FoxM1), MUC4, CDX2, Cyclin D1, Cyclin A2, AHSP, Bcl-2, survivin, VEGF, PGK1, CA9, MMP2, MMP9, CDX2, PD-L1, miR155-3p, miR-146a, p53	proliferation, survival, migration, angiogenesis, anti-tumor immune suppression	[70, 80, 82–86, 89–91, 93, 96, 98, 99]
ECs	Cyclin D1, Bcl-3, bFGF, VEGF-C, HIF-1α, miR-21, c-jun, eNOS, ICAM-1, NOSTRIN	proliferation, survival, migration, angiogenesis	[71, 80, 82, 85, 88, 92, 97, 123]
Stem cells	Stat1, Socs2, Socs3, Cish, Ifnar2, Irf9, Alkaline phosphatase (ALP), Myc, Sox2	differentiation, migration, self renewal	[62, 84, 95]
Other cells	Bfgf, Pax, Igf2bp2, Hmga1, TIMP1, siR-199a, SHP-1	migration, angiogenesis, inflammation	[81, 88, 113, 121, 193]

### Other potential mechanisms of STAT3-regulated angiogenesis

A genome-wide ChIP-assay in macrophages and T cells found that STAT3 binding sites are widely distributed [100, 101]. STAT family members collaborate to regulate transcription. For example, STAT1 often binds to chromatin in a STAT3 dependent manner (Figure 2B). However, in T cell-mediated anti-tumor immunity, STAT3 activation inhibits immune responses by antagonizing NF-κB and STAT1-mediated expression of T helper 1 (Th1) cytokines [102, 103]. Although the antagonizing effect of STAT3 to STAT1 has been well documented [104], this effect has not been examined in ECs.

STAT3 requires co-factors such as Sp1 to promote gene expression [105] (Figure 2C). In addition, STAT3 can be acetylated due to interaction with CBP/p300 [62, 63, 91, 106]. Wu et al. demonstrated that nerve growth factor-induced gene B (NGFI-B or Nur77) promotes the acetylation of STAT3 by p300 while preventing the interaction between STAT3 and histone deacetylase 1 (HDAC1). Consequently, Nur77 is conducive to the expression of STAT3 target genes in the hypothalamus [107]. This suggests that STAT3 complex formation requires additional co-factors. The effect of Nur77 on STAT3 has not been reported in either cancer cells or ECs. Nur77 is involved in VEGF-A-induced angiogenesis [108], vascular hyperpermeability [109], and upregulation of integrin β4 expression in HUVECs [110]. Together, we speculate that Nur77 promotes angiogenesis by maintaining STAT3 activation.

STAT3 also inhibits transcription of a variety of genes. This is associated with DNA methylation as shown in Figure 2D. Acetylation of STAT3 at K685 is needed for its interaction with DNA (cytosine-5)-methyltransferase 1 (DNMT1) [94, 111]. SH-I-14 and resveratrol inhibit the acetylation of STAT3 (K685) and disrupt the interaction between STAT3 and DNMT1, which leads to de-methylation of tumor suppressor genes promoter regions in triple-negative breast cancer (TNBC) [94, 112]. STAT3 has been reported to repress tumor suppressor gene expression by binding to promoter regions of *p53*, *Src homology region2 domain-containing phosphatase-1 (SHP-1)*, and *Nr4a3* [96, 113, 114]. In tumor cells, p53 inhibits tumor angiogenesis under hypoxia conditions by promoting HIF-1α degradation and decreasing VEGF expression [115]. Mutations of *p53* at R175H, R273H, and R280K promote tumor angiogenesis [116]. SHP-1 is a key negative regulator of STAT3. In conjunction with DNMT1, STAT3 inhibits SHP-1 transcription by methylating the promoter region in T cell lymphomas [96]. Little is known about STAT3 associated epigenetic modification, and this needs further investigation regarding the mechanisms of STAT3-mediated angiogenesis.

**Figure 3:**
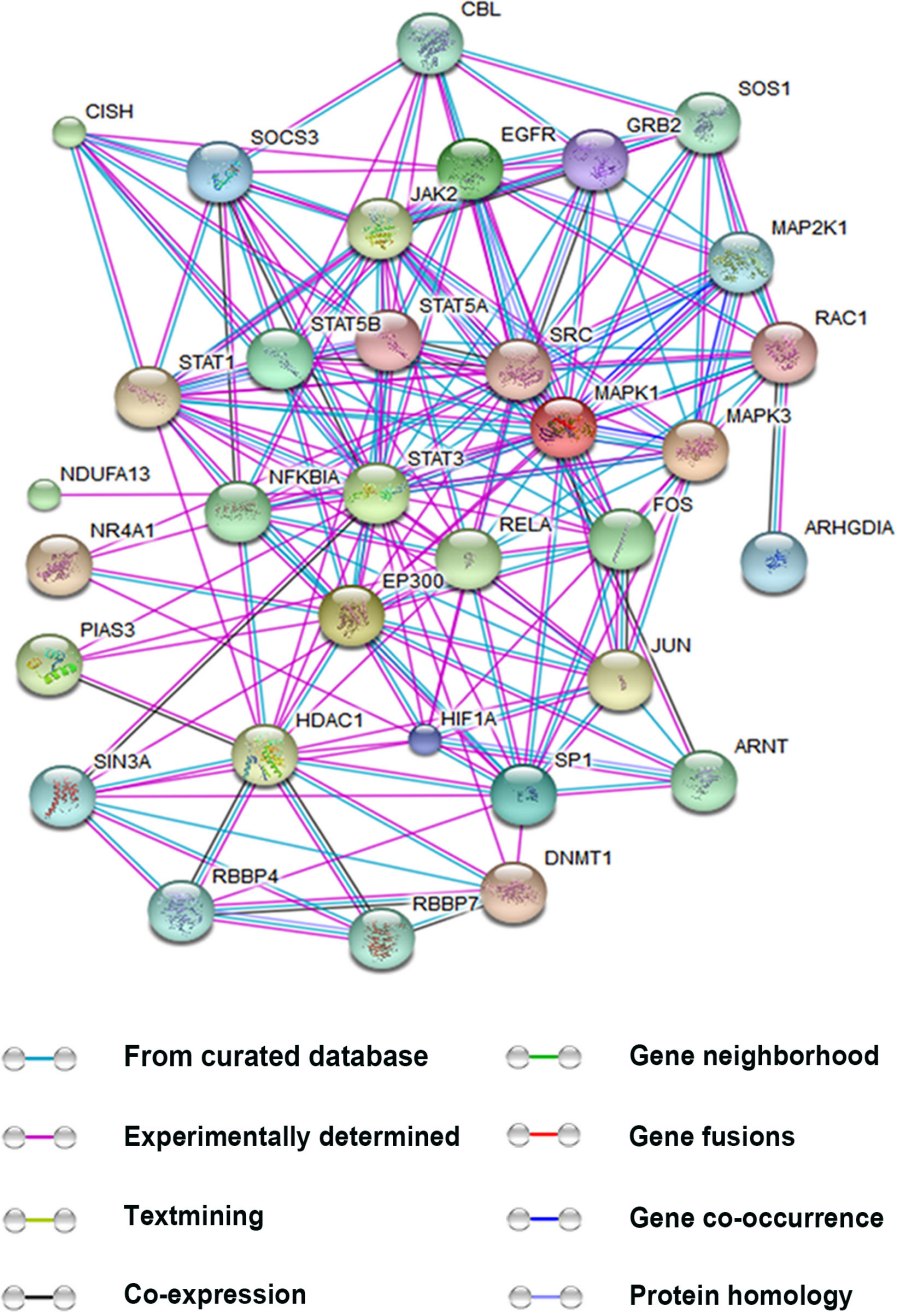
Interactions between STAT3 and other proteins This figure is generated using the website STITCH (http://stitch.embl.de/). The proteins were submitted to the human database. The interactions provided by the database are based on the eight aspects: known interactions encompassing results from curated databases, experimentally determined and predicted interactions gene neighborhood, gene fusions, and gene co-occurrence, textmining, co-expression, and protein homology. Only the proteins relevant to tumor angiogenesis and directly interplaying with STAT3 are shown. (ARNT, aryl hydrocarbon receptor nuclear translocator; CBL, Cbl proto-oncogene, E3 ubiquitin protein ligase; CISH, cytokine inducible SH2-containing protein; C-JUN, jun proto-oncogene; DNMT1, DNA (cytosine-5)-methyltransferase 1; EGFR, epidermal growth factor receptor; EP300, E1A binding protein p300; GRB2, growth factor receptor-bound protein 2; HIF-1α, hypoxia-inducible factor 1 subunit α; HDAC1, histone deacetylase 1; JAK, Janus kinase; MAPK1/3, mitogen-activated protein kinase 1/3; MAP2K1, mitogen-activated protein kinase kinase 1; NDUFA13, NADH dehydrogenase (ubiquinone) 1 alpha subcomplex 13; NIK, NF-κB-inducing kinase; NR4A1, nerve growth factor-induced gene B; PI3K, phosphatidylinositol 3-kinase; PIAS3, protein inhibitor of activated STAT3; RAC1, Ras-related C3 botulinum toxin substrate 1; RBBP4/7, retinoblastoma binding protein 4/7; RELA, v-relreticuloendotheliosis viral oncogene homolog A; SIN3A, SIN3 transcription regulator homolog A; SOCS, suppressors of cytokinesignaling; SOS1, son of sevenless homolog 1; STAT1/3/5A/5B, signal transducers and activators of transcription 1/3/5A/5B; SP1, specificity protein 1; SRC, v-src sarcoma (Schmidt-Ruppin A-2) viral oncogene homolog).

In addition to interacting with transcriptional co-factors, STAT3 has been reported to bind with many other proteins that may either promote or inhibit its activation. We utilized STITCH (http://stitch.embl.de/) to analyze proteins that interact with STAT3. Figure 3 shows the interaction networks of these proteins and also predicts their possible effects. Understanding the role of these interactions in angiogenesis will help shed light on the regulatory mechanism of STAT3.

### STAT3 in hypoxia-induced angiogenesis

**Figure 4:**
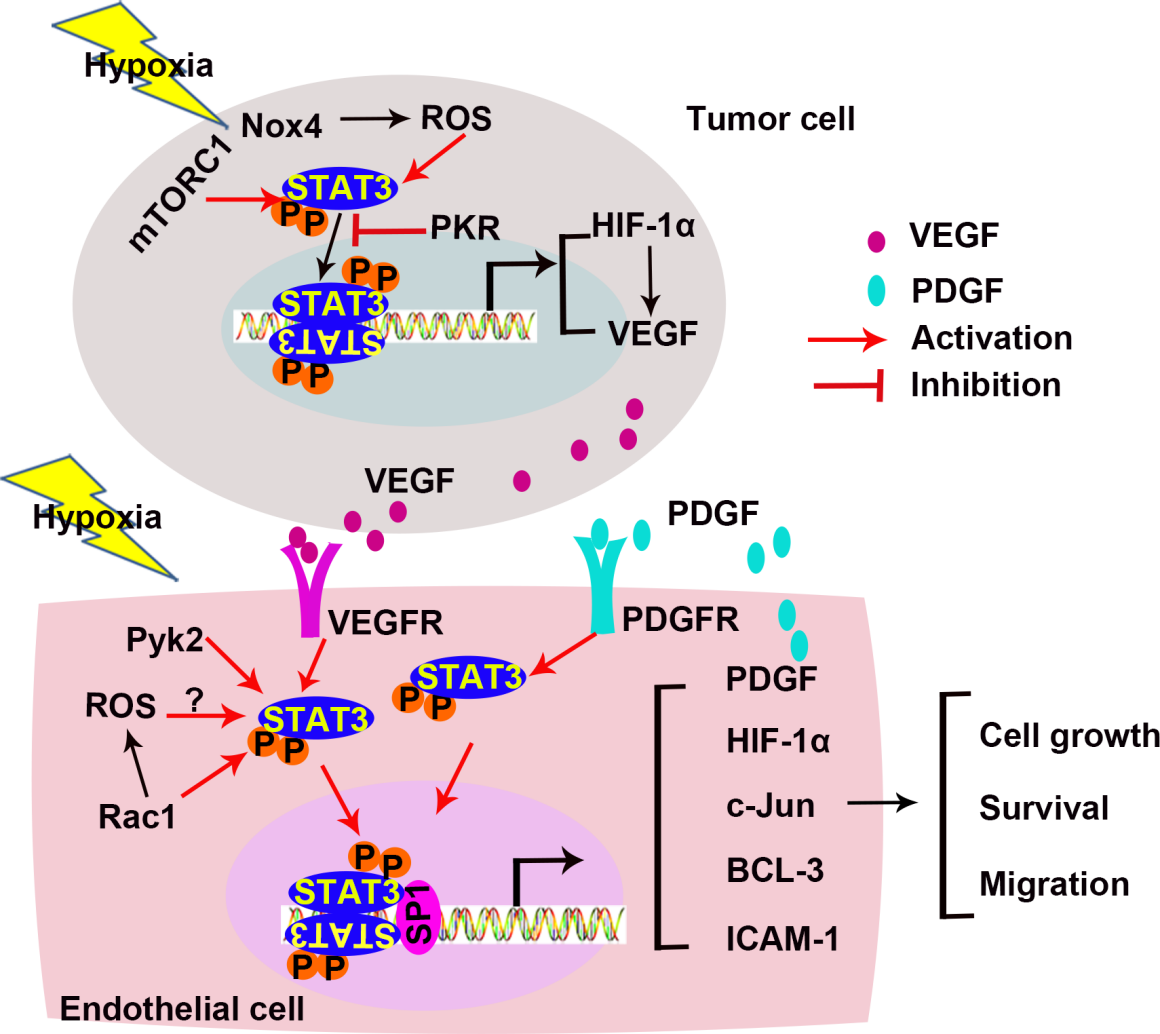
STAT3 in hypoxia-induced angiogenesis Hypoxia activates STAT3 in both tumor cells and ECs. Under hypoxic conditions, the STAT3/HIF-1α pathway promotes angiogenesis. Crosstalks between ECs and cancer cells also involve in angiogenesis. (ICAM-1, intercellular cell adhesion molecule-1; mTORC1, mammalian target of rapamycin complex 1; Nox4, nicotinamide adenine dinucleotide phosphate oxidase 4; PDGF-B, Platelet-derived growth factor-B; PKR, RNA-dependent protein kinase R; Pyk2, proline-rich tyrosine kinase 2; ROS, reactive oxygen species; Sp1, specificity protein 1).

Hypoxia is a common feature of cancer. It initiates a variety of cellular responses including activation of STAT3 and promotes expression of VEGF [64, 117]. The most crucial transcription factor activated under hypoxic conditions is HIF-1, which promotes hypoxia-inducible gene products, such as VEGF. HIF-1 contains two subunits, HIF-1α and HIF-1β. In normoxia condition, the expression of HIF-1β is sustained while HIF-1α is degredated in a HIF prolyl hydroxylases (PHDs) dependent manner. In hypoxia condition, the interaction of PHDs and HIF-1α is inhibited thus HIF-1α stability is maintained [118]. STAT3 and HIF-1α increase the expression of VEGF thus promote tumor angiogenesis. Figure 4 is a conceptual diagram that shows roles of STAT3 in hypoxia-induced angiogenesis in cancer cells and ECs.

During hypoxia, STAT3 and HIF-1α cooperatively activate HIF-1α target genes, VEGF, phosphoglycerate kinase 1 (PGK1), and carbonic anhydrase 9 (CA9) in prostate cancer cells [117]. As discussed before, the co-activators CBP/p300 and Ref-1/APE are required to promote the target genes expression. In cancer cells, the complexes containing STAT3, HIF-1α, CBP/p300, and Ref-1/APE bind to the *Vegf* promoter and increase VEGF expression [62]. In addition, STAT3 is required for both basal and induced expression of HIF-1α, and inhibits HIF-1α degradation [119]. Inhibition of STAT3 with a small-molecule inhibitor blocks HIF-1α and VEGF expression *in vitro* [85].

At hypoxia condition, ROS mediates STAT3 activation [120]. Nicotinamide adenine dinucleotide phosphate oxidase 4 (Nox4) is a major route of ROS production. Nox4 expression is up-regulated under hypoxia condition. Both Nox4 siRNA and ROS inhibitors decrease hypoxia-induced STAT3 activation. In addition, the conditioned medium from Nox4 knock-down tumor cells fails to induce tube formation of ECs *in vitro* [121]. In addition to ROS, several proteins affect STAT3 activity under hypoxia. By inhibiting STAT3, RNA-dependent protein kinase R (PKR), an eukaryotic initiation factor 2alpha (eIF2α) kinase, decreases HIF-1α and VEGF expression in cancer cells [122]. Mammalian target of rapamycin complex 1 (mTORC1) activates STAT3 during hypoxia in renal cystadenoma cells, and promotes HIF-1α and VEGF expression [123]. Together, STAT3 activation is crucial for HIF-1 α and VEGF expression in cancer cells under hypoxia.

Activation of STAT3 is also detected in ECs in response to abnormal oxygen supply. Hypoxia promotes human retinal microvascular endothelial cells (HRMVECs) migration, sprouting, and tube formation, mediated by increased expression of c-Jun via the proline-rich tyrosine kinase 2 (Pyk2)/STAT3 signaling pathway [124]. Hypoxia also up-regulates the transcriptional production of platelet-derived growth factor-B (PDGF-B) in ECs, resulting in STAT3 activation and the inhibition of EC apoptosis in an autocrine manner. Additionally, PDGF-B activates phosphatidylinositol 3-kinase (PI3K)/protein kinase B (AKT) pathway, the upstream of STAT3 activation [125]. Activation of STAT3 maintains the viability of hypoxia-treated endothelial colony-forming cells (ECFCs). Hypoxia activates STAT3 and increases the expression of a downstream gene, B-cell lymphoma 3-encoded protein (BCL-3) [126]. These results demonstrate that hypoxia activate STAT3 in distinct pathways in different cell types. No matter how the activation is initiated, the STAT3/HIF-1α pathway under hypoxic condition promotes angiogenesis.

### STAT3 in inflammation-induced angiogenesis

**Figure 5:**
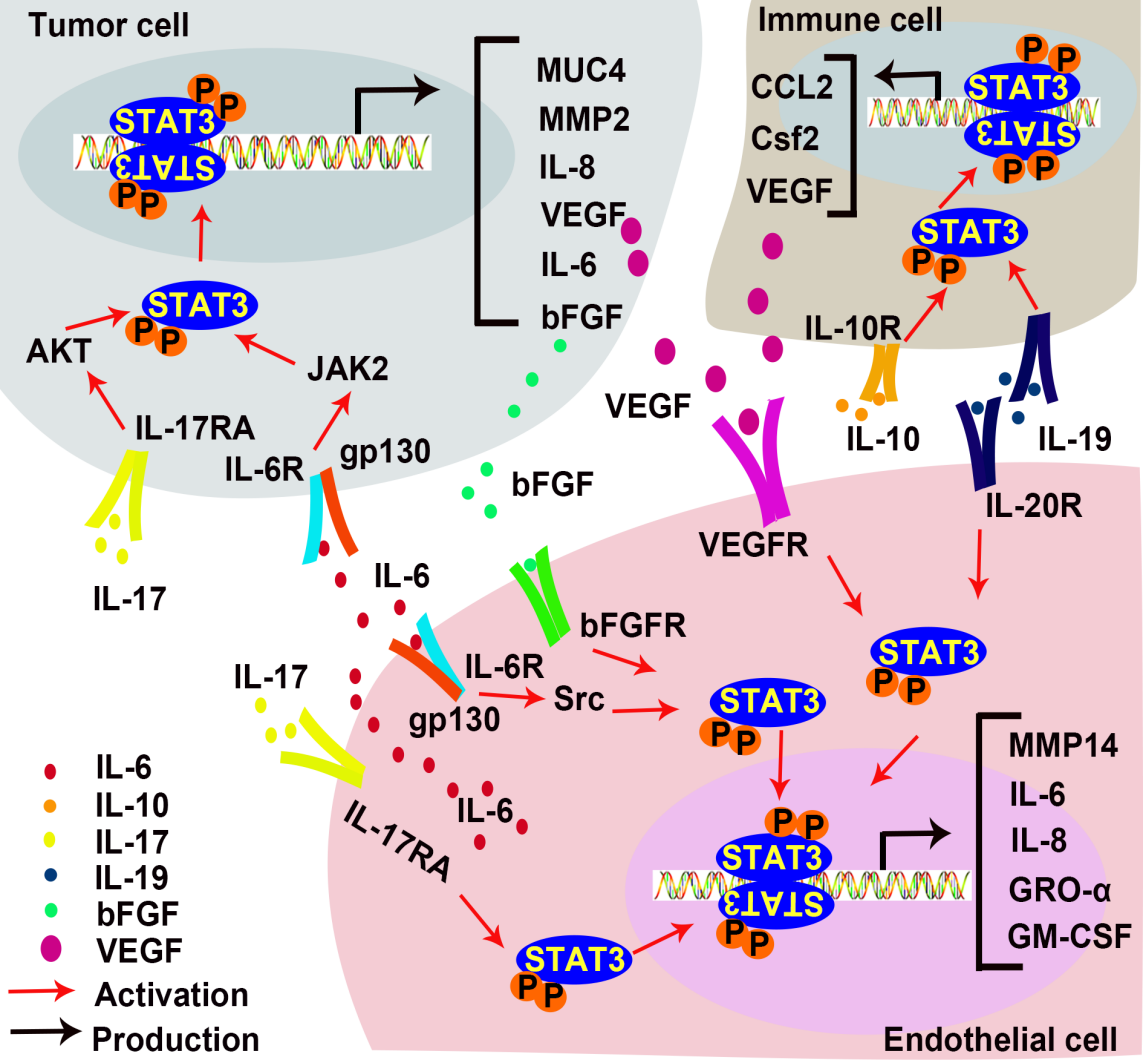
STAT3 in inflammation-induced angiogenesis IL-17 promotes IL-6 production in EC and tumor cells, and directly interact with ECs to promote angiogenesis. IL-6 activates STAT3 in tumor cells and ECs to increase expressions of bFGF and VEGF. IL-10 activates STAT3 in inflammatory cells, resulting in the up-regulation of VEGF. IL-19 promotes angiogenesis directly by activating STAT3 in EC and indirectly by stimulating macrophage-released pro-angiogenic factors. (AKT, Protein kinase B; bFGF, basic fibroblast growth factor; GM-CSF, Granulocyte-macrophage colony-stimulating factor; GRO-α also known as CXCL1, C-X-C motif chemokine ligand 1; IL-6, Interleukin 6; IL-10, Interleukin 10; IL-17, Interleukin 17; IL-19, Interleukin 19; JAK, Janus kinases; MMP2, metalloprotease 2; MMP14, metalloprotease 14; MUC 4, Mucin 4; VEGF, vascular endothelial growth factor).

Chronic inflammation markedly increases the risk of cancer [127]. It generates an inflammatory microenvironment to facilitate angiogenesis and support tumor progression [128]. An inflammatory microenvironment is characterized by the presence of specific inflammatory cells and inflammatory mediators [129]. Several studies suggest that STAT3 transmits inflammatory signals [98, 130–134]. Pro-inflammatory factors may activate STAT3 in both tumor cells and ECs to induce angiogenesis (Figure 5).

IL-6, a pleiotropic inflammatory cytokine, is a marker of chronic inflammation [135]. Many lines of evidence demonstrate that IL-6 promotes tumor progression in multiple ways [136–138], including enhanced angiogenesis [139] and lymphangiogenesis [140]. EC-derived IL-6 induces phosphorylation of STAT3 in tumor cells [141] and promotes tumor growth [142]. IL-6 treatment activates the gp130/STAT3 pathway in gastric cancer cells to up-regulate MUC4, which can be detected at early stages of the gastric carcinogenetic process. STAT3 binds to *Muc4* promoter at −123 to −115 and up-regulates its transcription [143, 144]. In basal cell carcinoma, IL-6 induces bFGF expression via the JAK/STAT3 pathway, promoting angiogenesis [145]. Competitive inhibition STAT3 with a dominant-negative mutant protein blocks IL-6-induced VEGF mRNA expression, and thus prevents VEGF-mediated angiogenesis and tumorigenesis. In contrast, inhibition of MAPK and PI3K in cervical cancer cells does not affect VEGF expression [146]. IL-6 also activates STAT3 in non-tumor cells. In murine lymphatic ECs, IL-6 induces VEGF expression via the Src-FAK-STAT3 pathway and increases lymphangiogenesis [147]. In chronic subdural hematoma (CSDH), an angiogenic and inflammatory disease which has an increased level of IL-6 in hematoma fluid, was found to activate STAT3 in ECs. This suggests that IL-6 enhances the JAK/STAT3 signaling pathway to promote EC proliferation and angiogenesis in CSDH outer membranes [148]. Moreover, human mesothelial cells in the peritoneal cavity of peritoneal dialysis (PD) patients have higher VEGF production due to IL-6-induced activation of STAT3 [149]. These results demonstrate that IL-6 promotes angiogenesis in STAT3-dependent manner by inducing VEGF expression in both tumor and non-tumor cells.

IL-17 increases IL-6 expression through the AKT pathway in hepatocellular carcinoma (HCC). The IL-17-induced IL-6 activates STAT3 and increases the expression of pro-angiogenic factors such as IL-8, MMP2, and VEGF to promote angiogenesis and tumor growth *in vivo* [150]. In both B16 melanoma and MB49 bladder carcinoma, IL-17-induced IL-6 production is mediated by STAT3 activation [151]. The increase of VEGF expression has also been detected in gastric cancer, which is stimulated by IL-17 in a STAT3 dependent manner [152]. Hou, et al. reported that IL-17 directly promotes human endothelial cells (HECs) activation and neutrophil recruitment by activating STAT3, thus up-regulating its downstream targets such as C-X-C motif chemokine ligand 1 (CXCL1 or GRO-α), granulocyte-macrophage colony stimulating factor (GM-CSF) and IL-8 [153].

Under conditions such as myocardial infarction (MI) and hypoxia, anti-inflammatory cytokines also promote angiogenesis via the STAT3 signaling pathway. IL-10 is a potent anti-inflammatory cytokine. Its effect on angiogenesis has been well documented [154–158]. IL-10 increases vascular density and improves ventricular function by activating STAT3 and increasing VEGF expression in the myocardium after MI [159]. Deletion of IL-10 impairs MI-induced endothelial progenitor cell (EPC) mobilization in mouse MI models. *In vitro*, IL-10 treatment increases EPC survival and augments EPC-mediated neo-vascularization via the activation of STAT3/VEGF signaling cascades [160]. IL-10 also activates STAT3 signaling in macrophages [161], and promotes pathological retinal angiogenesis by increasing VEGF expression under hypoxia [162]. Other studies of AMD patients suggest that IL-10 induces alternative activation of macrophages and vascular proliferation via the STAT3 signaling pathway, presumably by impairing SOCS3 feedback in senescent macrophages [163]. Other IL-10 family cytokines including IL-19 and IL-22 also facilitate angiogenesis in a STAT3-dependent manner [164, 165]. IL-19 promotes angiogenesis directly by activating STAT3 in vascular cells and indirectly by activating macrophages STAT3 to release pro-angiogenic factors [166]. Leukocytes secrete IL-22, which activates STAT3 in HCC and up-regulate VEGF expression [167]. Taken together, pro-inflammatory factors and anti-inflammatory factors both activate the STAT3 pathway. However, the target cells of these cytokines vary across different diseases. Intriguingly, STAT3 activation always promotes angiogenesis, regardless of the variations in upstream signaling events.

### STAT3 as a therapeutic target

**Figure 6:**
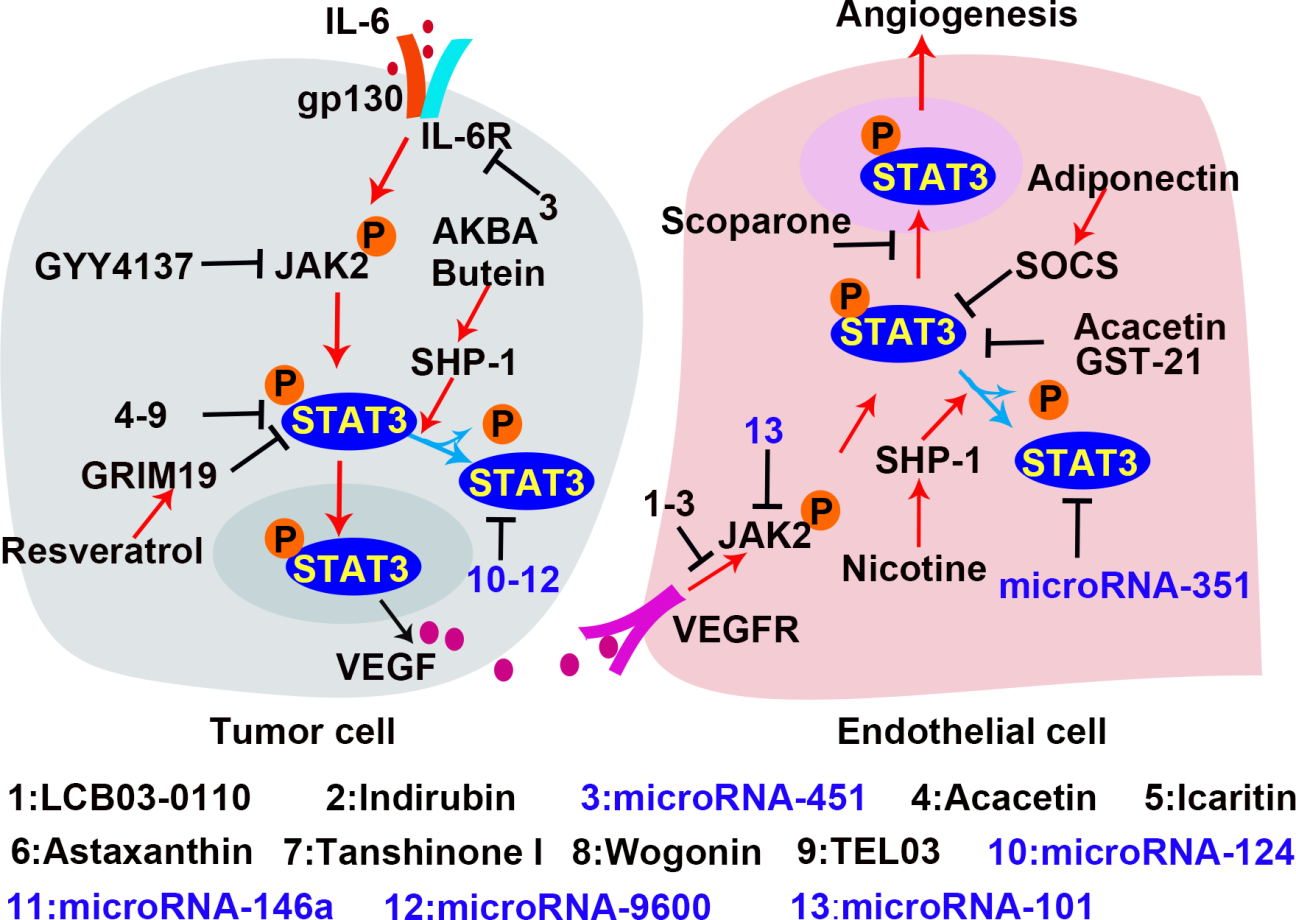
Inhibitors of the STAT3 signaling in tumor angiogenesis In the process of tumor angiogenesis, the rapid proliferation of tumor cells leads to local hypoxia and inflammation, which activate STAT3 in tumor cells to produce pro-angiogenic factor. VEGF is a potent pro-angiogenic factor to promote EC angiogenesis. The VEGF/VEGFR signal activates STAT3 which subsequently promotes endothelial cell proliferation and migration by regulating the transcription of the targeted genes. Inhibition of STAT3 is a potential therapeutic strategy for tumor growth and angiogenesis. The established inhibitors are shown with the indication of black “T” markers.

Inhibition of the STAT3 signaling is a potential therapeutic strategy for tumor and other angiogenesis related diseases [10, 19]. As shown in Tables 2 and 3 [85, 156, 159, 161–189], which summarize articles published since 2013, numerous natural and synthetic molecules are known to inhibit the components of the STAT3 signaling and related signaling pathways. The STAT3 molecules and pathways that are targeted by various inhibitors are illustrated in Figure 6.

**Table 2:** Inhibitors of STAT3 in development and angiogenesis

Inhibitors	Cell/Tissues	Mechanisms	References
*Natural*			
Scoparone	VSMC	Blocks the transportation of STAT3 from the cytosol to the nucleus	[169]
Indoxyl sulfate	Mouse EPCs/ human EPCs	Unclear	[182]
adiponectin	Human VSM and VECs	Induces SOCS to inhibit STAT3	[184]
melatonin	HepG2/HeLa/ HUVECs	Unclear	[186]
Tanshinone I	Vascular endothelial cells/ tumor	Inhibits STAT3 705 Tyr-phosphorylation	[85]
Indirubin	Human endothelial cells/ tumor	Inhibits VEGFR/JAK2/ STAT3 phosphorylation	[178]
Wogonin	Tumor/ HUVECs	Inhibits STAT3 705 Tyr-phosphorylation and promotes dephosphorylation	[172]
Astaxanthin	Tumor	Inhibit STAT3 phosphorylation	[170]
GYY4137	Human hepatocellular carcinoma (HCC)	Inhibits JAK2/STAT3	[195]
Icaritin	Renal cell carcinoma	Inhibits IL-6-induced STAT3 705 Tyr-phosphorylation	[191]
Butein	Human Multiple Myeloma/ tumor	Induces SHP-1 to dephosphorylate JAK2 and STAT3	[199]
resveratrol	Tumor	Induces GRIM19 to inhibit STAT3	[203]
Acacetin	HUVECs/ tumor	Inhibits STAT3 705 Tyr-phosphorylation	[187]
α -Solanine	Tumor	Unclear	[205]
JSI-124 (Cucurbitacin I)	Tumor	Unclear	[183]
*Synthetic*			
TEL03	Tumor	Inhibits STAT3 phosphorylation by interacting with SH2 domain	[190]
LCB03-0110	Human endothelial cells/ tumor	Targets VEGFR-2 kinase activity by binding to the ATP-binding site	[177]
P3971	Tumor	Unclear	[185]

**Table 3:** Inhibitory microRNAs for STAT3 in tumor development and angiogenesis

MicroRNAs	Tissues/ Cells	Mechanisms	References
27-nt from eNOS4th intron/Intronic microRNA	Human aortic endothelial cells (HAECs)	Inhibits eNOS and STAT3 expression	[175]
microRNA-124/ (let-7, miR-125, miR-26, or miR-101)	Ulcerative colitis/ tumor	Binds to STAT3 3′ UTR and reduces STAT3 expression	[174, 192]
MicroRNA-101	Pulmonary microvascular endothelial cells (PMVECs)	Targets JAK2	[197]
Mmu-microRNA-351	Mouse endothelial cells	Targets STAT3	[193]
MicroRNA-9600	Tumor/ Non–small-cell lung cancer (NSCLC)	Directly binds STAT3 and promotes degradation	[194]
MicroRNA-451	Hepatocellular carcinoma	Inhibits IL-6R	[196]
MicroRNA-539	Hepatocellular carcinoma	Unclear	[206]
MicroRNA-146a	Human cervical and colorectal cancer	Inhibits STAT3 expression	[86]
MicroRNA-133b and MicroRNA-135a	Human renal carcinoma	Unclear	[180]
MicroRNA-148a	Human gastric cancer	Inhibits cholecystokinin B receptor (CCK-BR)	[181]

Both natural materials and synthetic small molecules were developed to inhibit STAT3. Along with the increased understanding of microRNAs, a number of non-coding RNAs have been found to modulate the STAT3 signaling pathway. The most common inhibitors are designed to directly inhibit STAT3 expression and activation. For example, both tanshinone I and acacetin are natural materials that inhibit STAT3 activation by preventing STAT3 705 Tyr-phosphorylation in ECs and cancer cells. Treatment with these molecules down-regulates VEGF expression and attenuates vascular formation [85]. Astaxanthin, a non-provitamin A carotenoid with potent anti-oxidant effects, is predominantly found in microalgae, fungi, and plants [171]. Astaxanthin blocks STAT3 activity by forming hydrogen bond with Met 1428, Glu 1523, Arg 1593, and Asn 538 on STAT3 in tumor cells, abrogating cell proliferation, invasion, and angiogenesis [170]. Wogonin, a natural flavonoid [172], directly prevents the phosphorylation of STAT3 on tyrosine 705. Another natural product, scoparone, inhibits STAT3 by blocking its nuclear translocation and down-regulates MMP9, cyclinD, and survivin expression in VSMCs, which inhibits vascular remodeling [169]. In addition to natural materials, TEL03, a perylene derivative, is a dual inhibitor that targets both HIF-1α and STAT3 [190]. Surface plasmon resonance shows that TEL03 interacts with the residues of E612, S613, and E638 in SH2 domain of STAT3; therefore it disrupts the interaction between STAT3 and the phosphotyrosine-stimulating receptor. Icaritin, a hydrolytic product of icarin from a traditional Chinese herbal medicine (*genus pimedium*), restricts renal tumor growth by inhibiting STAT3 activity and VEGF expression *in vivo* [191]. As shown in Table 3, there are many microRNAs which reduce STAT3 expression by targeting STAT3 mRNA and impair tumor vascular formation. 27-nt from the eNOS 4th intron is an intronic microRNA which inhibits STAT3 expression in human aortic endothelial cells (HAECs) [175]. Other microRNAs such as microRNA-124 [173, 174, 192], microRNA-351 [193], and microRNA-146a [176] are also STAT3 inhibitors. Instead of binding to STAT3 mRNA, microRNA-9600 directly binds to STAT3 protein and promotes STAT3 degradation in NSCLC [194].

Inhibitors might also target components of the signaling pathway upstream of STAT3. LCB03-0110, a thienopyridine derivative, inhibits VEGFR-2 and c-Src kinase activity via preferential binding to the ATP-binding site. Consequently, STAT3 activation in ECs is inhibited, and angiogenesis is reduced [177]. Indirubin, an active constituent of banlangen [179], inhibits angiogenesis by blocking VEGFR2 phosphorylation on Tyr996 and Tyr1175, and inhibiting JAK/STAT3 signaling in ECs [178]. GYY4137, a hydrogen sulfide (H_2_S) donor, suppresses STAT3 activation by effectively reducing p-STAT3 levels in cancer cells. GYY4137 treatment leads to apoptosis and reduced release of angiogenesis factors in HepG2 cells [195]. MicroRNAs also block STAT3 upstream signaling to inhibit STAT3 activation. MicroRNA-451 inhibits IL-6R transcription by targeting IL-6R 3′ untranslated region (UTR), reducing IL-6R/STAT3 signal and decreasing VEGF secretion in HCC [196]. MicroRNA-101 inhibits JAK2 transcription by targeting JAK2 3′UTR [197], and inhibits STAT3 activation in ECs to abrogate vascular formation.

SHP-1, SOCSs and protein inhibitor of activated STAT (PIAS3) are negative regulators of STAT3 and become the targets for suppression of STAT3 activity [198]. AKBA is a boswellic acid isolated from *boswellia serrata*, and butein has been isolated from numerous plants. Both AKBA and butein inhibit STAT3 via SHP-1 in human multiple myeloma [199, 200]. Resveratrol is abundant in red grapes with no reported toxicity towards normal cells at effective anticancer doses [201]. PIAS3 is up-regulated by resveratrol treatment and inhibits STAT3 activation in cancer cells [202]. In addition to increasing PIAS3, resveratrol also up-regulates genes associated with retinoid-IFN-induced mortality 19 (GRIM19) to inhibit STAT3. Previous studies demonstrated that GRIM19 down-regulates p-STAT3 and that inhibiting GRIM19 with siRNAs restores cell proliferation induced by resveratrol [203, 204]. In addition, inhibitors such as α-Solanine [205] and microRNA-539 [206] are demonstrated to be effective inhibitors of STAT3 activity and tumor growth. However, the mechanisms behind these effects remain to be investigated.

## CONCLUSIONS AND PROSPECTS

Angiogenesis is required for tumor development, and inhibition of angiogenesis is a promising strategy for tumor treatment. Angiogenesis is a complex process that is mediated by multiple factors, among which STAT3 is one of the most prominent transcription factors. Hypoxia and inflammatory factors activate STAT3 to promote tumor angiogenesis. Persistent activation of STAT3 contributes to HIF-1α and VEGF expression in cancer cells and other non-ECs. VEGF then activates STAT3 in ECs, and promotes cell proliferation, migration, and survival. In addition, STAT3 inhibits the expression of tumor suppressors, such as the anti-angiogenesis transcription factor p53.

Although many studies have demonstrated the positive role of STAT3 in promoting tumor angiogenesis, further research is needed to illuminate the detailed mechanisms of STAT3 in this process. For instance, it is worth noting that members of the STAT family are highly conserved, and STAT1, STAT5 and STAT6 also participate in vascular formation. Current studies focused on the effects of STAT3 homodimers in angiogenesis, but STAT3 might also form heterodimers with STAT1 or STAT5. The interactions between these STATs in regulating angiogenesis need to be furtherly investigated. Treatment strategies involving the inhibition of STAT3 activity have been investigated and specific agents are demonstrated to be effective. Nevertheless, treatments with single agent have not achieved an optimal effect due to the complexity of STAT3 regulation *in vivo*. The identification of more selective agents and combination use of the targeted inhibitors will be important challenges for development of therapeutic strategies.

## Abbreviations

AKT: protein kinase B
AMD: age-related macular degeneration
BCL-3: B-cell lymphoma 3-encoded protein
Bcl-xL: B-cell lymphoma-extra large
bFGF: basic fibroblast growth factor
CA9: carbonic anhydrase 9
CRC: colorectal cancer
CBP/p300: CREB-binding protein
ChIP: Chromatin immunoprecipitation
CNS: central nervous system
CPCs: cardiac progenitor cells
CSDH: chronic subdural hematoma
CXCL1 or GRO-α: C-X-C motif chemokine ligand 1
CypD: cyclophilin D
DNMT1: DNA (cytosine-5)-methyltransferase 1
ECs: endothelial cells
ECFCs: endothelial colony-forming cells
ECM: extracellular matrix
eIF2α: eukaryotic initiation factor 2alpha
EGF: epidermal growth factor
EGFL7: epidermal growth factor (EGF)-like domain 7
EPC: endothelial progenitor cell
EPO: erythropoietin
ESCs: embryonic stem cells
ERK1/2: extracellular signal-regulated kinase 1/2
EZH2: enhancer of zeste homolog 2
FAK: focal adhesion kinase
FRalpha: folate receptor alpha
GAS: interferon-gamma activated sequence
gp130: glycoprotein 130
GM-CSF: granulocyte-macrophage colony stimulating factor
GPI: Glycophosphatidyl inositol
GRIM19: genes associated with retinoid-IFN-induced mortality 19
H_2_S: hydrogen sulfide
HAECs: human aortic endothelial cells
HAT: histone acetyltransferase
HCC: hepatocellular carcinoma
HDAC1: histone deacetylase 1
HECs: human endothelial cells
HIF-1α: hypoxia inducible factor 1alpha
hPMSCs: Human placenta-derived multipotent mesenchymal stromal cells
HUVECs: human umbilical vein endothelial cells
HRMVECs: human retinal microvascular endothelial cells
IFN-α: interferon-α
IL-1β: interleukin-1β
IL-6: interlaukin-6
IL6R: IL-6 receptor
I/R: ischemia/reperfusion
JAK2: Janus kinases 2
KDa: kilo-dalton
L1CAM: L1 transmembrane glycoprotein
LPS: lipopolysaccharide
MAPK: mitogen-activated protein kinase
MEF: mouse embryonic fibroblasts
MI: myocardial infarction
MMPs: matrix metalloproteinases
MSCs: mesenchymal stem cells
mTORC1: Mammalian target of rapamycin complex 1
NF-YA: Nuclear transcription factor-Y alpha
NGFI-B or Nur77: nerve growth factor-induced gene B
NLS: nuclear localization signal
Nox4: Nicotinamide adenine dinucleotide phosphate oxidase 4
NSCLC: non-small-cell lung cancer
OSM: Oncostatin M
PEP: phosphoenolpyruvate
PD: peritoneal dialysis
PDGF: platelet-derived growth factor
PKC: protein kinase C
PKM2: pyruvate kinase M2
PHDs: prolyl hydroxylases
PI3K: phosphatidylinositol 3-kinase
PIAS3: protein inhibitor of activated STAT
PGK: phosphoglycerate kinase 1
PKR: RNA-dependent protein kinase R
Pol II: RNA polymerase II
PRC2: polycomb repressive complex 2
Pyk2: proline-rich tyrosine kinase 2
Ref-1/APE: redox effector factor-1/apurinic/apyrimidinic endonuclease
RECK: reversion-inducing cysteine-rich protein with kazal motifs
RTKs: receptor tyrosine kinases
ROS: reactive oxygen species
SH2: Src homology 2
SHP-1: Src homology region2 domain-containing phosphatase-1
Sp1: specificity protein 1
SOCS3: suppressor of cytokine signal 3
STAT3: Signal transducer and activator of transcription factor 3
Th1: T helper 1
TNBC: triple-negative breast cancer
uPAR: urokinase receptor
TNF-α: tumor necrosis factor-α
UTR: untranslated region
VEGF: vascular endothelial growth factor
VSMCs: vascular smooth muscle cells
